# Physiological and gut microbiome changes associated with low dietary protein level in genetically improved farmed tilapia (GIFT, *Oreochromis niloticus*) determined by 16S rRNA sequence analysis

**DOI:** 10.1002/mbo3.1000

**Published:** 2020-03-16

**Authors:** Hao‐jun Zhu, Jun Qiang, Yi‐fan Tao, Tlou Kevin Ngoepe, Jing‐wen Bao, De‐ju Chen, Pao Xu

**Affiliations:** ^1^ Wuxi Fisheries College Nanjing Agricultural University Wuxi China; ^2^ Key Laboratory of Freshwater Fisheries and Germplasm Resources Utilization Ministry of Agriculture Freshwater Fisheries Research Center Chinese Academy of Fishery Sciences Wuxi China

**Keywords:** GIFT, growth, gut microbiome, low‐protein diet, physiological parameters

## Abstract

The aim of this study was to determine the effects of different dietary protein levels on the growth, physiological parameters, and gut microbiome of genetically improved farmed tilapia (GIFT, *Oreochromis niloticus*). Two pellet feed diets with low (25%, LPD) and normal (35%, NPD) protein levels were fed to GIFT in aquaria at 28°C for 8 weeks. The LPD reduced trypsin activity and inhibited the growth of GIFT. The serum alanine amino transferase and aspartate transaminase activities, hepatic malondialdehyde content, and superoxide dismutase, glutathione peroxidase, and catalase activities were significantly higher in LPD GIFT than in NPD GIFT (*p* < .05). The LPD led to decreased lysozyme activity and increased levels of C3 (*p* < .05). A 16S rRNA gene profiling analysis showed that the LPD significantly affected the gut microbial composition. Compared with the NPD, the LPD significantly decreased intestinal microbial diversity (*p* < .05). The macronutrient distribution affected the taxonomic profile of gut bacteria, mainly the phyla Bacteroidetes, Proteobacteria*,* and Firmicutes*.* The LPD favored growth of the genus *Bacteroides.* The NPD appeared to increase the abundance of the genera *Lawsonia, Romboutsia,* and *Sphingomonas*. Our results showed that, compared with NPD GIFT, the LPD GIFT had weakened nonspecific immune function, altered microbial community structure, and decreased gut microbial diversity.

## Graphical abstract

Two types of diets with low and normal protein levels were fed to genetically improved farmed tilapia. The results suggest that insufficient dietary proteins are likely to restrict growth and weaken disease resistance. The pellet feed diets with low protein level strongly affected the gut microbial composition and significantly decreased intestinal microbial diversity.

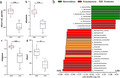

## 1 INTRODUCTION

Dietary protein is a key factor determining the growth rate of animals, but it is also the most expensive component of balanced pellet feed (Jones, Silva, & Mitchell, 1996; Qian, Cui, Xie, & Xue, 2002). The total protein requirement depends on the fish species, its life stage, and the digestibility and amino acid composition of the protein source. Excess protein supplied in the diet is metabolized as an energy source, and this results in increased production of nitrogenous waste material. When such wastes accumulate in water, they can be harmful to fish growth and the water environment (Catacutan & Coloso, 1995; Tibbetts, Lall, & Anderson, 2000). If the protein level in feed is too low, the fish cannot achieve their optimal growth rate (Abdel‐Tawwab, Ahmad, Khattab, & Shalaby, 2010; Hanley, 1991; Shiau & Lan, 1996), and their survival decreases (Eguia, Kamarudin, & Santiago, 2010; Péres, Zambonino Infante, & Cahu, 1998). According to previous reports, the protein requirement of tilapia with different specifications is 30%–50% of feed weight (Abdel‐Tawwab et al., 2010; Ng & Romano, 2013; Siddiqui, Howlader, & Adam, 1988).

Many microorganisms inhabit the intestinal tract of animals and play vital roles in maintaining the balance of the intestinal environment and the health of the host (Björkstén, 2010). The composition of intestinal microbes is relatively stable (Gorbach, Nahas, Lerner, & Weinstein, 1967) and host specific (Costello, Gordon, Secor, & Knight, 2010; Li, Yu, Feng, Yan, & Gong, 2012). Intestinal microorganisms are related to the development of natural immunity and adaptive immunity (Jiao & Wang, 2013). They participate in carbohydrate and protein metabolism, promote the absorption of mineral elements such as iron and magnesium, and participate in the synthesis of many vitamins and nonessential amino acids (Li, Sun, & Wu, 2017). Thus, the gut microflora has far‐reaching impacts on the nutrition, physiology, and immunity of the host. The colonization and homeostasis of intestinal microorganisms are very important for the host animal. Many studies have explored differences in gut microbiota among different age groups of animals and identified core gut bacteria in human and model organisms. The life cycle and growth environment of aquatic animals are more diverse than those of land animals. Accordingly, the intestinal microbiota is more diverse in aquatic organisms than in terrestrial animals (Ni, Yu, Zhang, & Gao, 2012; Xiong et al., 2017).

Genetically improved farmed tilapia (GIFT, *Oreochromis niloticus*) have many beneficial characteristics including their stable genetic traits, fast growth rate, high fillet yield, and strong disease resistance. Consequently, tilapia is one of the main cultured species in China. Research has shown that the optimal protein demand of fish is much higher than that of land animals (Kaushik, Seiliez, Fracalossi, & Lall, 2010). The intensive fish culture industry is developing rapidly, and a shortage of high‐quality protein feed sources has become evident. Recently, low‐protein diets have become a hot topic in fish nutrition research. Indeed, low protein requirements contribute to maximized feed conversion efficiency, and feed cost is the main variable cost in fish production (Robinson & Li, 1997). Lower dietary protein levels may help to reduce nutrients entering aquatic ecosystems, which is one of the major negative impacts of aquaculture (Rui, Pousão‐Ferreira, & Oliva‐Teles, 2010). In this study, we determined the effect of different dietary protein levels on cultured GIFT. We analyzed the growth (body weight and body length), physiological parameters, and gut microbiome composition of GIFT fed with pellet feed containing 35% and 25% protein. These results provide new information for developing protocols to produce healthy GIFT as a market commodity and for breeding.

## 2 MATERIALS AND METHODS

### 2.1 Materials

Methanesulfonate (MS‐222) was purchased from the Sigma Chemical Company. The TIANamp Stool DNA Kit (DP328) was obtained from the Tiangen Biotech Co., Ltd. The liver and intestinal biochemical detection kits were purchased from the Jian Cheng Bioengineering Institute (Nanjing, China).

### 2.2 Animals

Healthy juvenile fish were obtained from the Freshwater Fishery Research Center of the Chinese Academy of Aquatic Sciences (Yixing, China). Before the experiment, the fish were stored separately in indoor plastic drums containing dechlorinated water at 28°C ± 0.5°C, under a 12‐hr light–12‐hr dark cycle for 1 week. At this stage, GIFT were accustomed to receiving commercial sub‐combined feed (crude protein 35.0%, crude fat 8.0%).

### 2.3 Diet formulation

According to the nutritional requirements of tilapia, we designed and produced fish feed for this experiment. In accordance with previous studies (Abdel‐Tawwab et al., 2010; Ng & Romano, 2013), we established diets with 35% and 25% protein as the normal‐protein diet (NPD) and the low‐protein diet (LPD), respectively. The composition of these feeds is shown in Table 1. There was no significant difference in the levels of conventional nutrients, crude fat, and total energy between the NPD and LPD (*p* > .05).

**Table 1:** Composition and proximate analyses of experimental diets

	Dietary lipid level (g/kg dry diet)	
	LPD	NPD
Fish meal^a^	50	50
Casein	40	134
Gelatin	10	33.5
Corn starch	378	260.5
Soybean oil	60	60
Soybean meal	120	120
Cottonseed meal	150	150
Rapeseed meal	150	150
Vitamin premix^b^	10	10
Mineral premix^c^	10	10
Choline chloride	5	5
Vitamin C phosphate ester	2	2
Ca(H_2_PO_4_)_2_	15	15
Total	1,000	1,000
Crude protein	25.16	35.09
Crude lipid	6.88	6.92
Gross energy (KJ/g diet)	1705.20	1689.22

a American Seafood, purchased from Coland Feed Co., Ltd., Wuhan, PR China. Chemical composition: moisture: 4.26%; crude protein: 68.97% of dry matter; crude lipid: 8.97%; ash: 12.15%.

b Vitamin premix (mg/kg dry diet):V_A_ 10, V_D_ 0.05, V_E_ 400, V_K_ 40, V_B1_ 50, V_B2_ 200, V_B3_ 500, V_B6_ 50, V_B7_ 5, V_B11_ 15,V_B12_ 0.1, V_C_ 1,000, inositol 2000, choline 5,000.

c Mineral premix (mg/kg dry diet): FeSO_4_·7H_2_O 372, CuSO_4_·5H_2_O 25, ZnSO_4_·7H_2_O 120, MnSO_4_·H_2_O 5, MgSO_4_ 2,475, NaCl 1,875, KH_2_PO_4_ 1,000, Ca(H_2_PO_4_)_2_ 2,500.

### 2.4 Experimental design

In total, 240 fish were separated into two experimental groups of 120 according to body weight. They were randomly allocated to four plastic drums (1.5 m^3^) containing 1 m^3^ aerated tap water. Each drum was equipped with a submersible pump for water circulation and filtration. Fish were fed with the experimental diet at 7:00, 11:30, and 16:00 hr every day. The amount of diet was about 5% of GIFT body weight and was increased or decreased depending on the residual bait the previous day. We checked the feeding and swimming of GIFT at each feeding time to monitor injuries. Any remaining feed was removed 30 min after feeding. Feces were siphoned daily from the bottom of the drums, and half of the water was replenished every 2 days. A 12‐hr light–12‐hr dark cycle was maintained during this 8‐week feeding experiment.

### 2.5 Growth performance analysis and sample collection

At the end of the experiment, the GIFT were fasted overnight and then harvested. To avoid effects of stress on the various measurement indexes, the fish were anesthetized by immersion in 1% MS‐222 before being killed. Eight fish were collected from each drum (32 samples per group), and their body weight and length were measured. Before dissection, two samples from each drum (8 samples per group) were chosen at random, and blood for hematological analyses was extracted from the tail blood vessel of each anaesthetized fish with an air‐dried 2‐ml syringe. Blood samples were centrifuged at 4,000 *g* for 20 min at 4°C and then stored at −80°C until serum analysis. A necropsy was performed, and liver (0.2 g) and intestine (5 cm piece anterior to the anus) tissues were collected, frozen in liquid nitrogen, and stored at −80°C until analysis.

### 2.6 Liver and intestinal biochemical analyses

The liver biochemical analyses included malondialdehyde (MDA, mmol/L) content and activities of superoxide dismutase (SOD, mg/L), glutathione peroxidase (GSH‐PX, mg/L), and catalase (CAT, mg/L). The intestinal biochemical analyses included α‐amylase (AMY, mg/L) and trypsin (TRY, mg/L) activities. These analyses were conducted using kits from the Jian Cheng Bioengineering Institute. All kits contained corresponding standards to validate the assays.

### 2.7 Blood biochemical analysis

Serum was analyzed using an automatic biochemical analyzer (BS400, MINDAR) to determine total protein (TP, g/L), total cholesterol (TC, mmol/L), and triglyceride (TG, mmol/L) contents, as well as the activities of alanine aminotransferase (ALT, U/L) and aspartate transaminase (AST, U/L). Reagents and test kits were purchased from MINDRAY. Complement C3 (C3, mg/L) and lysozyme (LYZ, ng/L) were detected using kits from the Jian Cheng Bioengineering Institute. All test kits contained the corresponding reference materials to verify the analytical results. These kits were used strictly in accordance with the manufacturer's instructions.

### 2.8 DNA extraction and 16S rRNA gene sequencing

DNA was extracted from 16 samples (eight per group) using the TIANamp stool DNA kit (DP328). The PCR amplifications of the 16S ribosomal RNA (rRNA) gene and library preparation were performed by the LC‐Bio Technology Co., Ltd. The V3–V4 region of the prokaryotic (bacterial and archaeal) small‐subunit (16S) rRNA gene was amplified with slightly modified versions of primers 338F and 806R, under the following PCR conditions: 98°C for 30 s, followed by 35 cycles at 98°C for 10 s, 54°C for 30 s, and 72°C for 45 s, and final extension at 72°C for 10 min. The libraries were sequenced on the 300PE MiSeq Illumina sequencing platform.

### 2.9 Statistical analyses

Samples were sequenced on the Illumina MiSeq platform according to the manufacturer's recommendations, by LC‐Bio. Paired‐end reads were merged using FLASH. Under specific filtering conditions, FQTRIM (v.0.94) was used to filter the original tags to obtain high‐quality clean tags. Chimeric sequences were filtered using Vsearch (v. 2.3.4). Sequences with similarities ≥97% were assigned by Vsearch (v.2.3.4) to the same operational taxonomic unit (OTU). Four indexes were calculated to evaluate the α‐diversity of each sample: Chao1, Observed species, Shannon's index, and Simpson's index. These indexes were calculated using QIIME (v. 1.8.0). Drawing rarefaction curve with R Software (v. 2.15.3).To assess differences in species complexity between samples, a β‐diversity analysis was used. The β‐diversity and principal coordinate analyses (PCoA) were conducted using QIIME. All data are presented as mean ± standard deviation (*SD*) unless indicated otherwise. Differences in nutrient composition between diets were analyzed by Student's *t* test and were considered significant at *p* < .05. The Wilcoxon rank‐sum test was used to determine the significance of differences in α‐diversity and abundance of phyla among samples. Two‐tailed Mann–Whitney *U* tests were also conducted. Correlations between α diversity and physiological parameters were analyzed using Spearman's *r* correlation analyses. Significance of the PCoA was estimated using ADONIS. In all analyses, differences were considered significant at *p* < .05.

## 3 RESULTS

### 3.1 Growth of GIFT

During the experiment, the animals did not exhibit abnormal behavior. The average body weight and length are shown in Table 2. The final body weight and body length were greater for NPD GIFT than for LPD GIFT (*p* < .05).

**Table 2:** Mean body weight and body length of GIFT fed NPD versus LPD

Diet	Body weight (g)		Body length (mm)	
	Initial	Final	Initial	Final
NPD	0.81 ± 0.06	58.52 ± 6.56^b^	ND	114.80 ± 4.26^b^
LPD	0.83 ± 0.07	23.80 ± 4.02^a^	ND	88.40 ± 4.77^a^

Note

Student's *t* test, *p* < .05.

Abbreviation: ND, not detectable. 1

### 3.2 Biochemical parameters of GIFT

The hepatic antioxidant capacity and the activities of digestive enzymes in the intestine are summarized in Table 3. The hepatic MDA content and the activities of SOD, GSH‐PX, and CAT were significantly lower in the NPD fish than in the LPD fish (*p* < .05). Compared with the LPD fish, the NPD fish showed higher TRY activity (*p* < .05). The serum parameters are shown in Table 3. The activities of AST and ALT in serum were significantly higher in the LPD fish than in the NPD fish (*p* < .05). Compared with fish in the LPD group, those in the NPD group showed significantly higher C3 and LYZ levels (*p* < .05).

**Table 3:** Biochemical parameters of GIFT fed with NPD versus LPD

Index	NPD	LPD	*p*‐value^a^
Liver parameters (*N* = 8) (10% Homogenate)			
MDA (mmol/L)	59.52 ± 4.46	74.10 ± 4.77	.04
SOD (mg/L)	34.74 ± 2.17	47.39 ± 1.89	.00
GSH‐PX (mg/L)	12.91 ± 0.63	15.47 ± 0.54	.01
CAT (mg/L)	15.77 ± 0.86	19.01 ± 0.71	.01
Intestinal parameters (*N* = 8) (10% Homogenate)			
AMY (mg/L)	119.60 ± 3.81	109.90 ± 3.92	.09
TRY (mg/L)	57.17 ± 1.47	51.09 ± 1.38	.01
Serum parameters (*N* = 8)			
C3 (mg/L)	361.02 ± 23.76	248.72 ± 18.26	.00
LYZ(ng/L)	36.80 ± 2.02	27.23 ± 2.77	.01
ALT (U/L)	15.34 ± 4.31	30.75 ± 3.57	.00
AST (U/L)	82.40 ± 13.18	157.38 ± 23.72	.00
TP (g/L)	23.75 ± 2.00	22.92 ± 2.68	.44
TC (mmol/L)	2.16 ± 0.21	2.00 ± 0.21	.10
TG (mmol/L)	0.58 ± 0.10	0.63 ± 0.14	.31

a Student's *t* test, *p* < .05.

### 3.3 Metadata and sequencing

**Figure 1:**
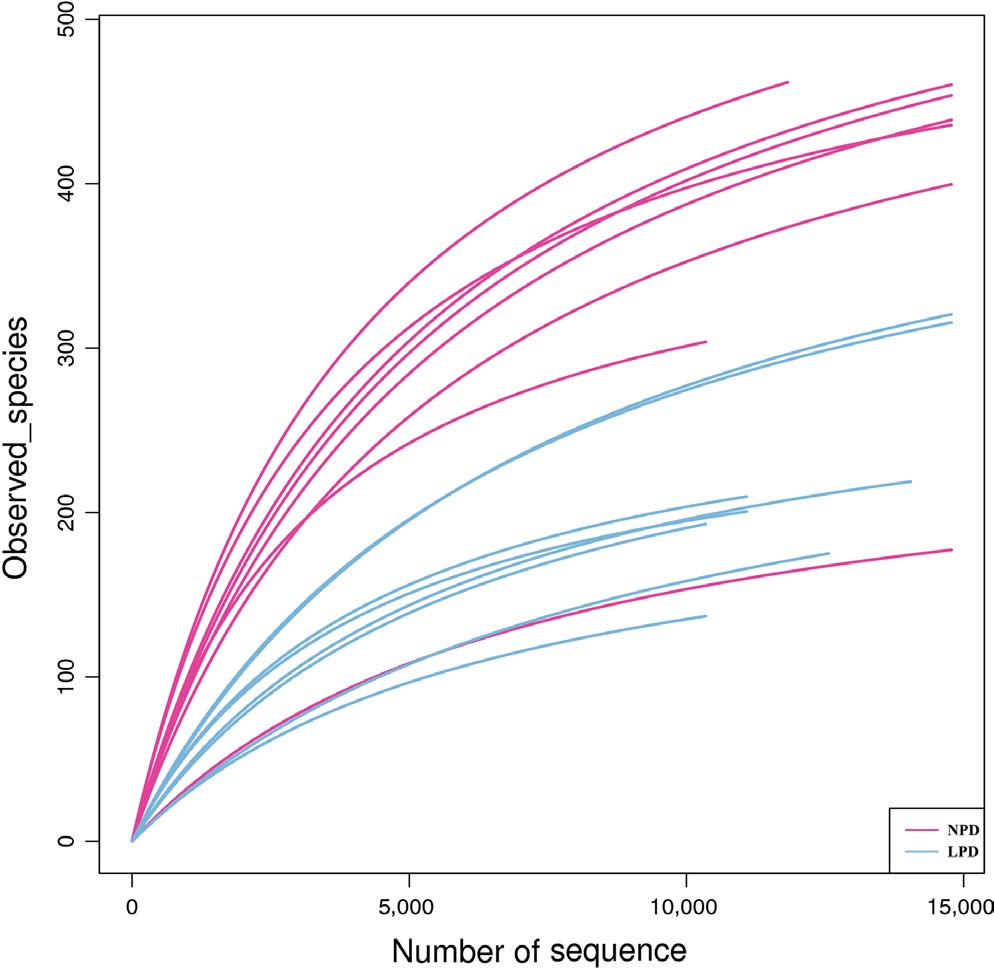
Rarefaction curves and estimators of different samples

Sixteen samples (eight NPD, eight LPD) were collected for sequencing, and 291,639 reads were assigned to 1,561 nonsingleton OTUs after OTU picking and chimera checking. Each sample had 350 OTUs and 36,455 sequences on average. The rarefaction curves and estimators are shown in Figure 1. The curve shows that the sequencing depth of intestinal microflora in each sample was fully captured, so all samples were suitable for further analysis.

### 3.4 LPD effects on gut microbial α and β diversities

**Figure 2:**
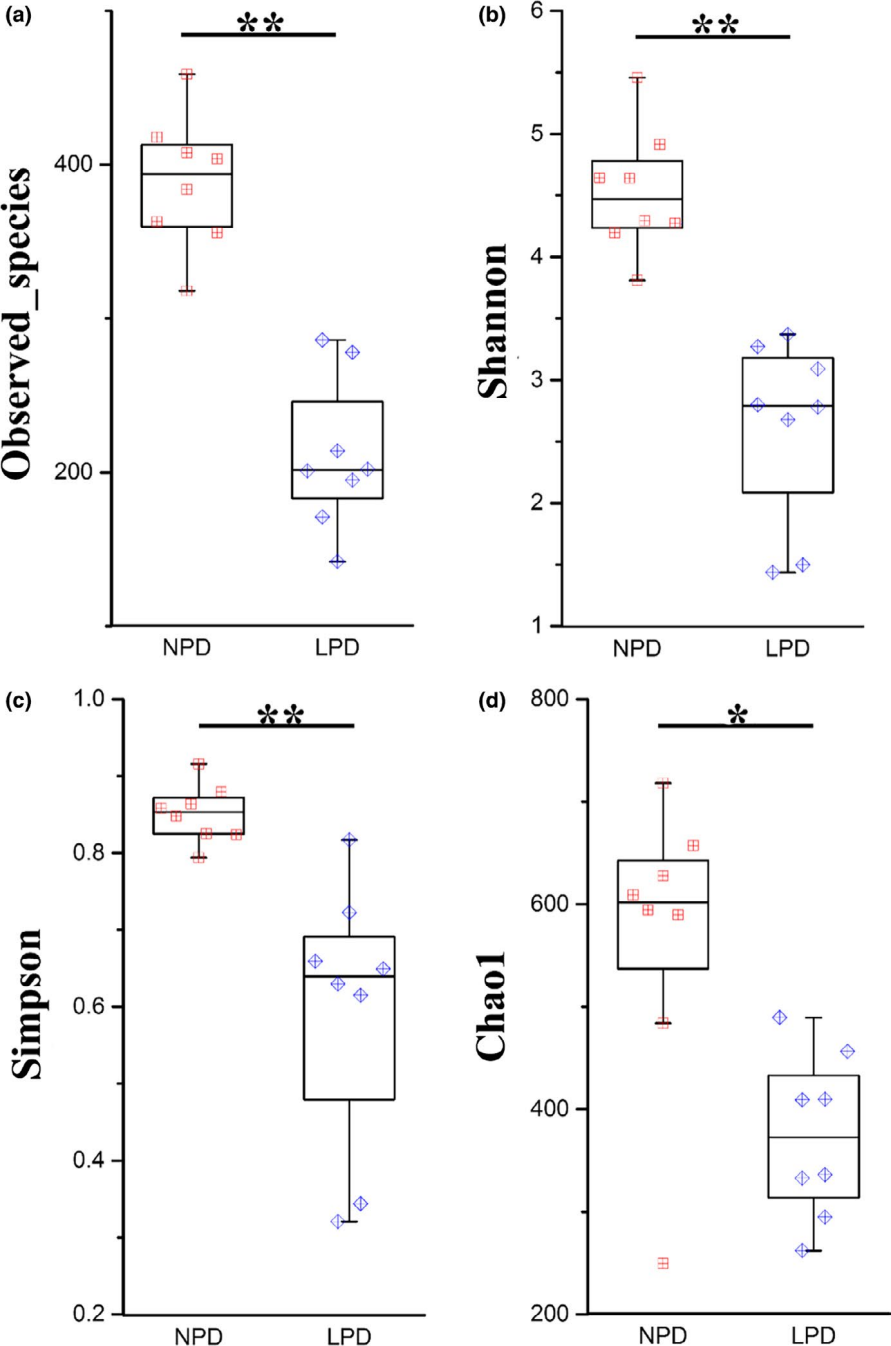
Measures of alpha diversity for normal‐protein diet (NPD) and low‐protein diet (LPD) genetically improved farmed tilapia (GIFT). (a) Observed species, (b) Shannon's index, (c) Simpson's index, (d) chao1 index. Red, NPD GIFT; blue, LPD GIFT. * and ** indicate significant difference in abundance between dietary groups (Mann–Whitney *U* test, *:*p* < .05, **:*p* < .01)

Four α‐diversity indexes were calculated: observed species (Figure 2a), Shannon's diversity index (Figure 2b), Simpson's diversity index (Figure 2c), and Chao1 (estimated OTUs) (Figure 2d). These indexes represented the richness and diversity of the microbiota. The α‐diversity in the intestine was higher in the NPD fish than in the LPD fish (*p* < .05).

**Figure 3:**
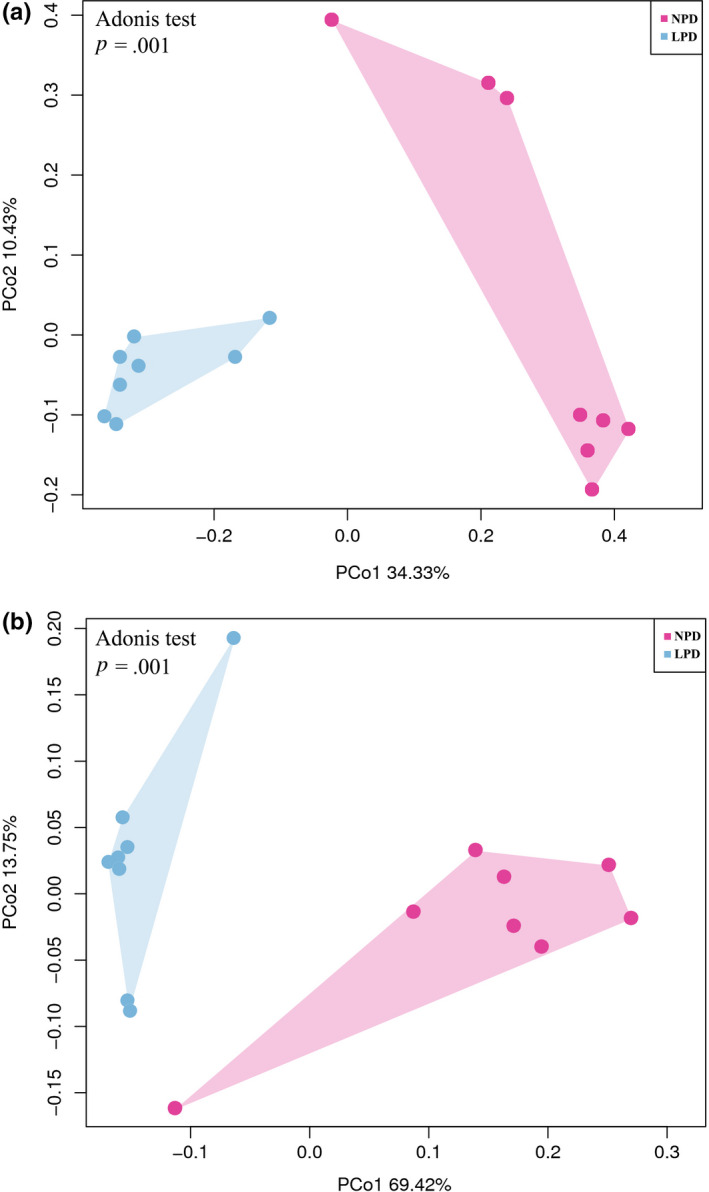
16S rRNA gene surveys showing effects of diet on gut microbial community. Bacterial beta diversity analysis based on principal coordinate analysis (PCoA) of unweighted (a) and weighted (b) and UniFrac matrices. Percentage of variation explained by principal coordinates (PC1 and PC2) is indicated on axes. Significance of the data was estimated using ANOSIM

Spearman's *r* correlation analyses (Table 4) showed that Shannon's and Simpson's indexes were correlated with C3. We also evaluated β diversity to quantify differences in microbial community composition between the NPD and LPD groups. The PCoA method based on weighted and unweighted single fractal distance matrices was used to study the relationship between samples based on intestinal microbial community structure. On the PCoA plot shown in Figure 3, each symbol represents the gut microbiota of a sample. This plot showed that the microbiota composition of NPD fish was significantly different from that of LPD fish (ADONIS analysis, *p* = .001).

**Table 4:** Spearman's r correlation coefficient between α diversity and physiological parameters in GIFT fed with NPD versus LPD

Index	Observed_species		Shannon's		Simpson's		Chao1	
	*r*	*p*‐value	*r*	*p*‐value	*r*	*p*‐value	*r*	*p*‐value
MDA	−.041	.880	−.094	.729	−.131	.628	.121	.656
SOD	−.371	.158	−.461	.073	−.312	.239	−.403	.122
GSH‐PX	−.144	.594	−.250	.350	−.197	.464	.053	.846
CAT	−.203	.451	−.082	.763	−.038	.888	.015	.957
AMY	.294	.269	.372	.156	.427	.099	.212	.431
TRY	.262	.327	.269	.313	.237	.377	.300	.259
C3	.479	.060	**.552**	**.027**	**.524**	**.037**	.282	.289
LYZ	.459	.074	.464	.071	.383	.143	.332	.208
ALT	−.397	.128	−.436	.092	−.358	.174	−.329	.213
AST	−.441	.087	−.344	.192	−.267	.318	−.159	.557
TP	.126	.641	.190	.481	.174	.520	.009	.974
TC	.419	.139	.458	.074	.330	.212	.418	.140
TG	−.050	.854	−.100	.712	−.069	.799	−.141	.602

Note

Positive values indicate positive correlations and negative values indicate inverse correlations between α diversity index and each physiological parameter. Significant values (*p* < .05) are indicated in bold font.

### 3.5 LPD effects on gut bacterial phyla

**Figure 4:**
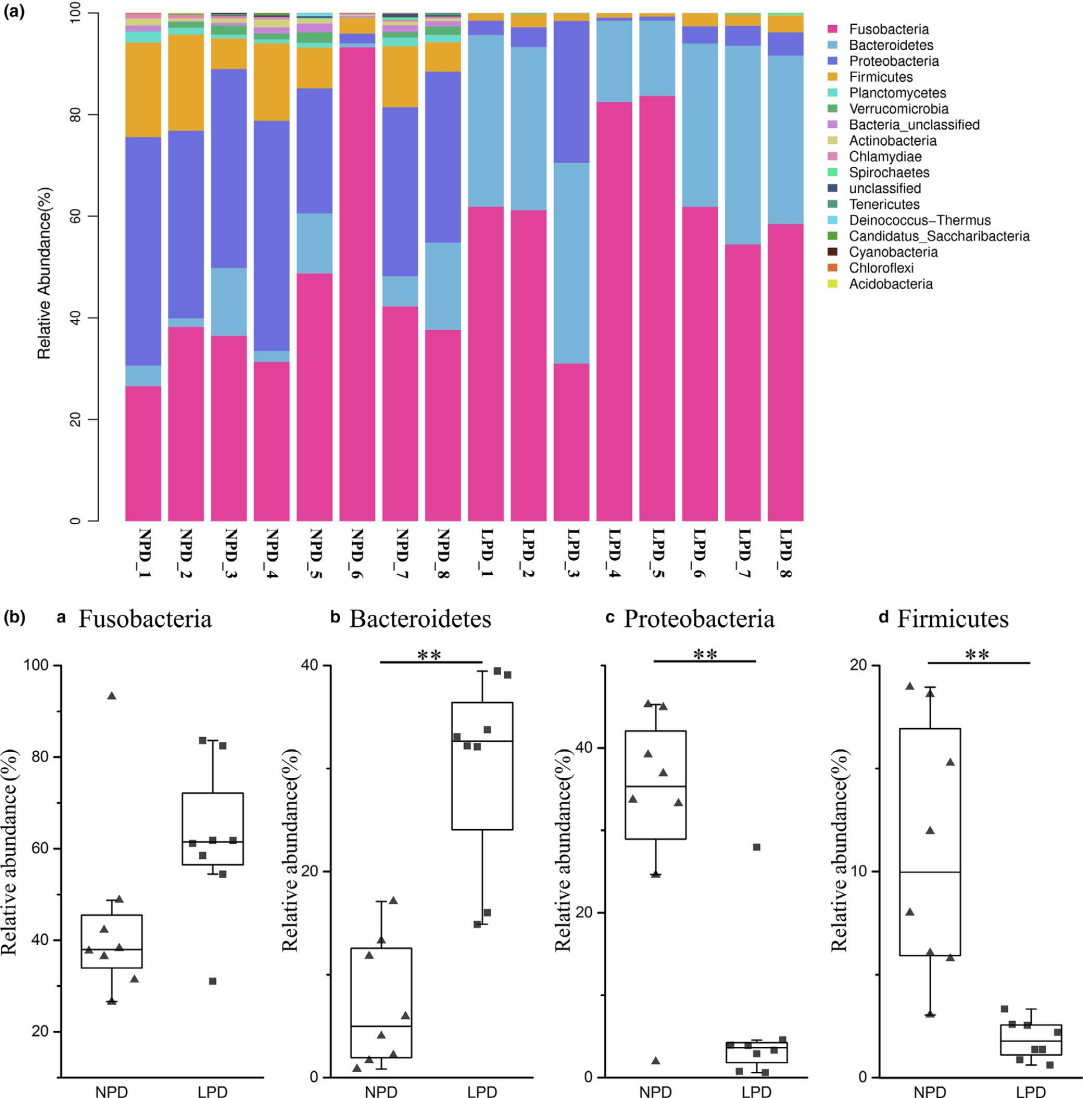
(A) Relative abundance of predominant taxa identified at phylum level. Each bar represents relative abundance in each sample; 20 most abundant taxa are shown. (B) Abundance of bacterial phyla (a–d) in normal‐protein diet (NPD) and low‐protein diet (LPD) genetically improved farmed tilapia (GIFT). Black lines in box plots represent medians of relative abundance. ** indicates significant difference in abundance between dietary groups (Mann–Whitney *U* test, **:*p* < .01)

We further analyzed the composition of intestinal flora at the phylum and genus levels. (Figure 4A). The relative abundance of the 20 richest OTUs in the intestinal microflora is represented by a cumulative column chart. Four bacterial phyla, Fusobacteria, Bacteroidetes, Proteobacteria*,* and Firmicutes, accounted for 53.09%, 18.60%, 19.24%, and 6.41% of all gut microbes, respectively. Bacteroidetes were more abundant, and Proteobacteria and Firmicutes were less abundant in the LPD fish than in the NPD fish (Figure 4Ba–d).

### 3.6 LPD effects on gut bacterial genera and species

**Figure 5:**
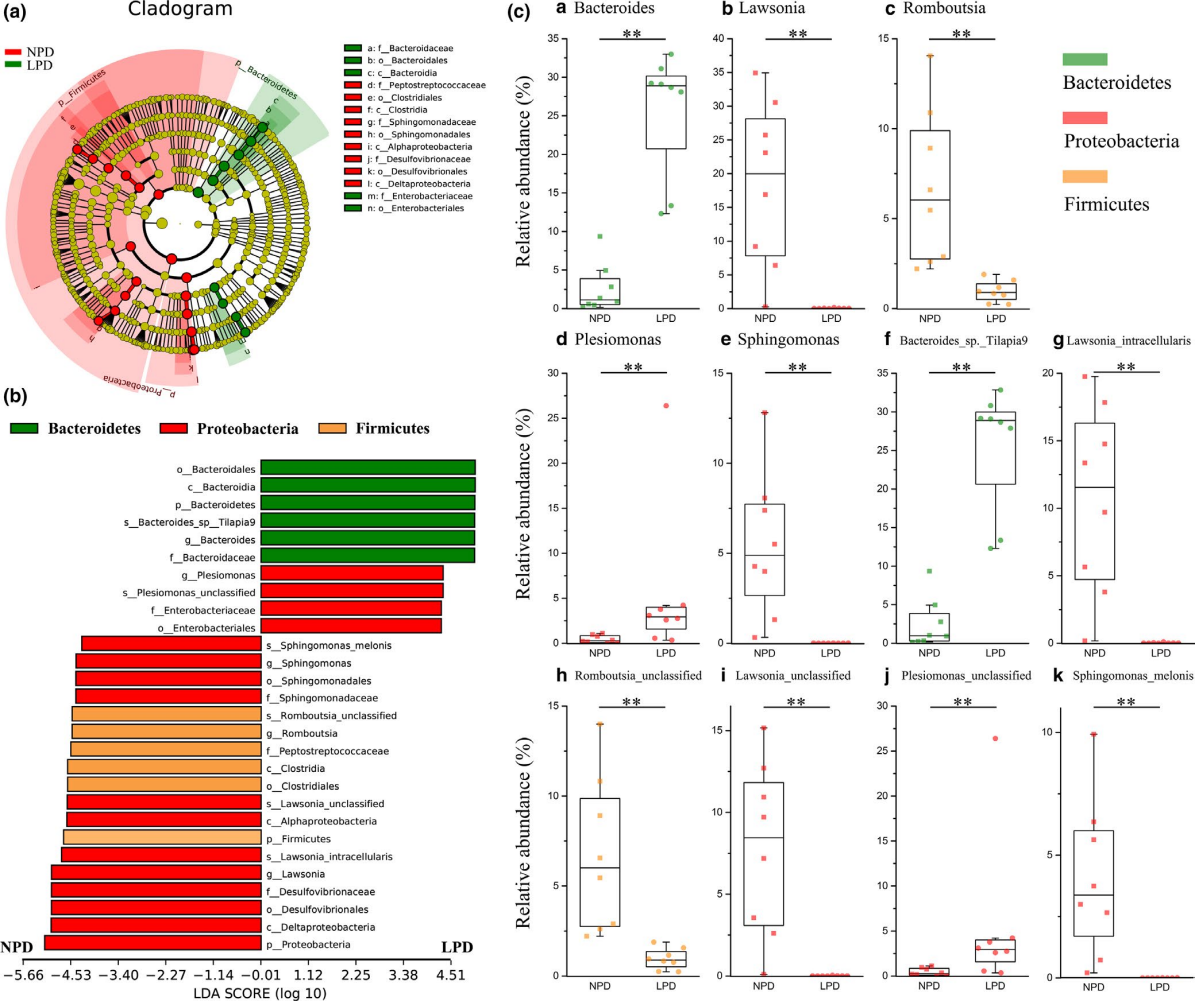
Linear discriminant analysis effect size (LEfSe) analysis comparing abundance of all detected bacterial taxa between genetically improved farmed tilapia (GIFT) fed with a normal‐protein diet (NPD) and those fed with a low‐protein diet (LPD). (A) Taxonomic cladogram produced from LEfSe analysis. Red and green indicate taxa enriched in NPG and LPD GIFT, respectively. Brightness is proportional to abundance of each taxon. (B) Taxa shown in histogram were determined to differ significantly in abundance between diets by Kruskal–Wallis test (*p* < .05) and have LDA score >4. Bacterial taxa associated with positive LDA scores (right) were overrepresented in LPD GIFT, and those with negative scores (left) were overrepresented in NPD GIFT. Green: bacteria in phylum Bacteroidetes; Red: bacteria in phylum Proteobacteria; Yellow: bacteria in phylum Firmicutes. (C) Abundance of selected bacterial genera (a–e) and species (f–k) in NPD and LPD GIFT. Black lines in box plots represent medians of relative abundance. Mann–Whitney *U* test was used to determine significance of differences between groups (**, *p* < .01)

The linear discriminant analysis (LDA) effect size (LEfSe) method was used to compare the abundance of all detected bacterial taxa between NPD and LPD fish. This method provides an estimate of the size of the effect and a *p*‐value for every comparison. Twenty‐eight bacterial taxa were identified as significant by both the Kruskal–Wallis test adjusted for multiple testing (*p* < .05) and the effect size analysis (LDA score > 4). Bacteroidetes were overrepresented in the LPD fish, and most Proteobacteria (75%) and all Firmicutes were overrepresented in the NPD fish (Figure 5B).

To identify which bacteria responded to differences in dietary protein levels, we calculated the relative abundance of selected bacterial genera and species (Figure 5C). At the genus level, *Bacteroides* was overrepresented, while *Lawsinia*, *Romboutsia,* and *Sphingomonas* were underrepresented in the LPD fish compared with the NPD fish (Figure 5Ca–e). *Bacteroides sp. Tilapia9* and *Plesiomonas* unclassified sp. were more abundant in LPD fish than in NPD fish, while *Lawsonia intracellularis*, *Romboutsia* unclassified sp.*,* and *Sphingomonas melonis* were overrepresented in NPD fish compared with LPD fish (Figure 5Cf–k).

## 4 DISCUSSION

### 4.1 LPD inhibited growth of GIFT

Many studies have shown that the growth rate and feed utilization of fish is related to the dietary protein level (Abdel‐Tawwab et al., 2010; Hafedh, 2010; Wang, Jiang, Ji, & Xie, 2011). In this 8‐week feeding trail, the final body weight and body length values were smaller for the LPD fish than for the NPD fish (*p* < .05). Hafedh (2010) found that the growth rate and feed efficiency of fish improved with increasing dietary protein levels. Thus, we speculate that a diet containing 25% protein is insufficient for GIFT (~0.8 g) and will severely inhibit their growth.

### 4.2 LPD altered serum parameters and antioxidant enzyme activities of GIFT

Normally, neuroendocrine regulation ensures that the contents of serum and liver biochemical components in fish remain relatively stable. Thus, these parameters can be used as indexes to evaluate the health status of fish. This study found that serum ALT and AST activities were increased in GIFT fed with a LPD (*p* < .05), indicating that endogenous metabolic transport was activated in these fish. In GIFT, AST and ALT are two main transaminases in hepatocytes. High AST and ALT activities generally indicate weakened or impaired liver function (Sheikhzadeh, Tayefi‐Nasrabadi, Oushani, & Enferadi, 2012). These results indicated that the LPD was probably harmful to the health of these experimental fish and may have increased the liver burden. Similar results were obtained in study on Black sea bream (*Sparus macrocephalus*) (Zhang et al., 2010).

In aquatic animals, LYZ is a critical component of the enzymatic system in hemolymph cells. A change in LYZ activity reflects a change in the nonspecific immunity level in organisms (Demers & Bayne, 1997). The complement system, which comprises about 35 proteins, is an important component of the innate immune system. The fish complement system can dissolve foreign cells and destroy them by phagocytic activity. Fish have a variety of complement protein subtypes, such as C3 (Holland & Lambris, 2002). We found that feeding with the LPD led to significant decreases in LYZ and C3 levels in GIFT (*p* < .03), indicative of poor immunity of these fish. This may be a stress response to malnutrition caused by the LPD. This result is similar to those reported in another study (Qiang, Yang, Wang, Xu, & He, 2013). In Spearman's r correlation analyses, we found that significant positive correlations between C3 and Shannon's index and Simpson's index (*r* = .552, *p* = .027, and *r* = .524, *p* = .037, respectively). Therefore, we speculate that the decrease in intestinal diversity may have led to a decrease in C3, which affected the immune system of GIFT.

The MDA level reflects the degree of oxidative damage in fish tissues (Jiang et al., 2016). Some of the critical antioxidant enzymes in fish are SOD, GSH‐Px, and CAT: SOD converts superoxide radicals into hydrogen peroxide, which is further scavenged by GSH‐Px and CAT (Jiang et al., 2016). In our experiment, hepatic MDA contents and the activities of SOD, GSH‐Px, and CAT were increased in the LPD group (*p* < .05), indicating that 8 weeks of the LPD was enough to activate the antioxidant system to remove excess free radicals. Similar results were obtained in a study on Nile tilapia (Yang et al., 2012). These results indicated that the nonspecific immunity of GIFT was affected by low dietary protein and that insufficient dietary protein affected disease resistance.

### 4.3 LPD changed the gut microbiome composition of GIFT

The intestinal tract is the main digestive part of fish. The dietary protein level can strongly affect the activity of enzymes that hydrolyze proteins and also affect TRY activity in fish (Chen et al., 2014). In this study, the activity of TRY was higher in the NPD fish than in the LPD fish (*p* < .05). This indicated that the digestion and absorption of nutrients were better, and consequently growth performance was better, in NPD fish than in LPD fish. In our study, dietary protein did not significantly affect AMY activity. Another study also found no significant difference in AMY activity in the gastrointestinal tract depending on protein levels in feed (Shao, Su, Xu, & Shu, 2004).

Intestinal microflora are a complex group of microorganisms that inhabit the gastrointestinal tract of fish. Microflora are closely related to many aspects of normal host physiology, from nutritional status to behavior and the stress response. In addition, intestinal microflora may be a central or contributing factor to many diseases (Icaza‐Chávez, 2013). Diet is the main factor affecting the composition and metabolism of intestinal microflora (Ringø et al., 2015). As mentioned above, four predominant bacterial phyla were identified in the GIFT in this study: Fusobacteria, Bacteroidetes, Proteobacteria*,* and Firmicutes. Fan et al. characterized microbial communities in the gut of intensively cultured GIFT during the peak breeding period and found that the dominant bacterial phyla were Proteobacteria, Actinobacteria*,* and Firmicutes (Fan et al., 2017). Similarly, Li et al. found that the dominant phyla in the gut of large yellow croaker (*Pseudosciaena crocea*) were Proteobacteria, Firmicutes, Fusobacteria, and Bacteroidetes (Li, Chen, & Song, 2017). It has been reported that Firmicutes are the dominant bacteria in the intestinal tract of most vertebrates (Ringø, Birkbeck, Munro, Vadstein, & Hjelmeland, 2010), but we found that the relative abundance of Firmicutes was only 10.96% (NPD GIFT) and 1.85% (LPD GIFT). The reported relative abundances of phyla vary among different studies. The reasons for these differences may be related to genotype/strain, diet, sex, age, growth environment, or even the sampling and analysis methods (Clements, Angert, Montgomery, & Choat, 2014; Deng & Swanson, 2014; Nayak, 2010).

The sequencing results showed that the LDP affected intestinal microbial diversity. According to the indexes of bacterial diversity, diversity was reduced in the LPD GIFT. Species diversity promotes stability and performance, so it is important in all ecosystems. Microbial diversity is an important indicator of body health (Fergus, 2010). Loss of intestinal biodiversity is associated with an increasing number of disease states. For example, inflammatory bowel disease (Frank et al., 2007). The intestinal tissues of animals raised under sterile conditions are not well developed, and their vascular, nutritional, and endocrine functions are also compromised. Compared with animals fed under normal conditions, those fed under aseptic conditions are more susceptible to infection and their gastrointestinal immune function is weaker (Ley, Peterson, & Gordon, 2006; Smith, Mccoy, & Macpherson, 2007). Therefore, higher diversity may be an important indicator of healthy microflora. In this study, the diversity of intestinal microflora was significantly higher in the NPD fish than in the LPD fish. The decrease in intestinal microbial diversity may have destabilized the intestinal microflora of the LPD fish and weakened their ability to combat disease.

The effect of diet on intestinal microflora in animals is becoming clearer, because several studies have shown that β‐diversity changes with dietary composition (David et al., 2014). In this study, we used weighted and unweighted UniFrac PCoA (Catherine & Rob, 2005), which rely on the phylogenetic divergence among the OTU, to analyze β‐diversity. We observed substantial differences in β‐diversity between the NPD and LPD groups, and each group had their own typical intestinal microflora. This phenomenon suggested that dietary protein levels can affect the intestinal microecological structure. These results also suggested that intestinal microflora may be affected by macronutrients, as observed in other animals affected by dietary changes.

The metabolic utilization of nutrients by intestinal microorganisms and their metabolites not only affect the utilization efficiency of feed nutrients, but also regulate the normal physiological functions of the host. We found that the most abundant phylum was Fusobacteria, most of whose members are obligate anaerobic Gram‐negative rods. The members of this phylum ferment carbohydrates or amino acids and peptides to produce various organic acids, such as acetic acid, propionic acid, butyric acid, formic acid, or succinic acid, depending on the bacterium and the substrate (Olsen, 2014).

Bacteroidetes is the most abundant group of Gram‐negative bacteria in the intestinal tract, and one of its main functions is to decompose polysaccharides (Salyers, 1984), which are related to body fat mass (BFM) content (Turnbaugh et al., 2006). Firmicutes are related to some physiological functions of the host organism. For example, members of the genus *Clostridium* can participate in the degradation of polysaccharides (Flint, Bayer, Rincon, Lamed, & White, 2008). Some studies have reported that Bacteroidetes and Firmicutes in intestinal flora are associated with obesity in humans and other animals (Bradlow, 2014; Ley et al., 2005; Turnbaugh et al., 2006), because they are involved in sugar metabolism, which is an important factor in obesity. Those studies reported that obese individuals had more Firmicutes than Bacteroidetes in their microflora. Thus, that particular combination of bacteria may be more efficient than other bacterial mixtures at intaking energy from a given food. Consistent with those results, our results also indicated that the abundance of Firmicutes was higher and that of Bacteroidetes was lower in NPD GIFT than in LPD GIFT. We speculated that the LPD fish may have a reduced ability to metabolize carbohydrates.

Among the Bacteroidetes, members of the *Bacteroides* genera were more abundant in LPD GIFT than in NPD GIFT. *Bacteroides* is the main genus in the lower intestinal tract, and molecular interactions among these species can influence host functions, for example, development of the immune system. If bacteria escape from the intestine into the peritoneal cavity due to trauma, they can cause life‐threatening infections including bacteremia (Balows, 2002). Thus, the LPD GIFT with higher relative abundance of *Bacteroides* may be at higher risk of diseases caused by *Bacteroides* spp. Among the Firmicutes, members of the genus *Romboutsia* were more abundant in NPD GIFT than in LPD GIFT. Some members of *Romboutsia* have probiotic activity. For example, the probiotic activity of a *Romboutsia* species was found to be associated with intestinal changes that alleviated acute pancreatitis in rats (Gerritsen, 2015). It has been reported that C3 is related to the abundance of *Romboutsia* (Wu, 2018). In this study, Spearman's r correlation analyses also confirmed a correlation between C3 and intestinal microbial diversity. Further studies should analyze the effect of dietary protein on the abundance and activity *Romboutsia*.

Proteobacteria is the largest branch of prokaryotes, accounting for the vast majority of known gram‐negative bacteria. This group includes a wide variety of pathogens, such as *Escherichia*, *Salmonella*, *Vibrio*, *Helicobacter*, *Yersinia*, and *Legionellales* (Brock, 2000). We detected two species belonging to this phylum, *L. intracellularis* and *S. melonis,* in the GIFT in this study. *Lawsonia intracellularis* is an important animal pathogen, particularly in pigs, and it causes the disease syndrome proliferative enteropathy (Mcorist, Gebhart, Boid, & Barns, 1995). This specific intracellular bacterium has been identified as the cause of many unrelated intestinal diseases in animals and birds. (Smith & Lawson, 2001). However, this bacterium is entirely dependent upon its host to facilitate replication and infection, which result in the disease syndrome. Further research is needed to determine whether *L. intracellularis* will cause illness or not in GIFT. The metabolic capacity of *Sphingomonas* has been utilized to provide important commercial benefits in biotechnology. For example, these organisms can degrade some refractory pollutants (White, Sutton, & Ringelberg, 1996). Unfortunately, they can also readily degrade the copper pipes that transport drinking water and cause diseases in animals and plants. *Sphingomonas melonis* was identified as the pathogen responsible for brown spots on yellow Spanish melon fruits (Roberto et al., 2002). There are few reports of this bacterium in the microbiota of fish. Therefore, it is unknown whether this bacteria is an opportunistic pathogen in the intestine of GIFT.

## 5 CONCLUSION

Our results showed that a LPD affected TRY activity in the gut, reduced the growth of GIFT, and altered serum parameters (AST, ALT), antioxidant enzyme activities (MDA, SOD, GSH‐PX, CAT), and immune capacity (LYZ, C3). Together, the results suggested that insufficient dietary protein are likely to restrict growth and weaken disease resistance. The LPD strongly affected the intestinal microbial composition of GIFT. Compared with NPD fish, LPD fish showed significantly decreased intestinal microbial diversity. The distribution of macronutrients in the different diets affected the composition of intestinal microflora, primarily the phyla Bacteroidetes, Proteobacteria*,* and Firmicutes*.* The LPD favored the growth of *Bacteroides,* while the NPD resulted in increased abundance of the genera *Lawsonia, Romboutsia,* and *Sphingomonas*. Further research is needed to clarify the complex relationships among diet, intestinal microorganisms, and host metabolism. When using LPDs for cultured fish, we need to pay attention to the energy levels and balance of amino acids to prevent damage.

## CONFLICT OF INTEREST

None declared.

## AUTHOR CONTRIBUTIONS

Zhu Haojun took the lead in data curation, formal analysis, and writing‐original draft; Jun Qiang equally contributed to conceptualization and took the lead in supervision; Yi‐Fan Tao made the supporting role in data curation, formal analysis, and writing‐original draft and equally contributed in writing‐review & editing; Kevin Ngoepe made the supporting role in writing‐original draft and writing‐review & editing; Jing‐wen Bao made the supporting role in project administration, writing‐original draft, and writing‐review & editing; De‐ju Chen made the supporting role in writing‐original draft and writing‐review & editing; Pao Xu equally contributed to conceptualization and took the lead in project administration.

## ETHICS STATEMENT

Animal utilization has been approved by the Freshwater Fisheries Research Center (FFRC, CAFS) of the Chinese Academy of Aquatic Sciences and carried out under its supervision.

## Data Availability

The raw sequence data are available at https://www.ncbi.nlm.nih.gov/bioproject/PRJNA528151.

## Footnotes

1 The different lowercase superscripts mean significant differences (*p* < 0.05) in mean body weight and body length of GIFT fed NPD versus LPD.
